# A Novel Intraoral Optical Scan-Transfer Device for Full-Arch Implant Reconstruction

**DOI:** 10.3390/dj13030134

**Published:** 2025-03-19

**Authors:** Cemal Ucer, Rabia Sannam Khan, Gwyn Jones

**Affiliations:** 1 ICE Postgraduate Institute, University of Salford, Manchester M50 3XZ, UK; ucer@icedental.institute; 2 RDT, Creative Dental Solutions (CIS) Dental Laboratory, Manchester M50 3XZ, UK; gwyn@creativeimplantsolutions.com

**Keywords:** intraoral optical scan-transfer, full jaw implant reconstruction, dental implantology, analogue techniques

## Abstract

**Background**: Dental implantology has undergone significant advancements with the integration of digital workflows, transforming the processes of planning, designing, surgical delivery, and prosthetic rehabilitation. Among these innovations, intraoral optical scanning (IOS) has emerged as a preferred method over traditional analogue impressions. This preference is due to its cost-effectiveness, efficiency, and streamlined patient-friendly use while producing clinically acceptable results in terms of trueness and precision, particularly for short-span implant prostheses. **Methods**: However, the clinical utility of intraoral scanning is significantly affected by the lack of reference points and difficulties in moisture and bleeding control at the time of immediate implant placement surgery in the fully edentulous arch. Current evidence supports the general consensus that the traditional analog impression technique still provides superior trueness and precision compared to IOS, specifically in full-arch implant cases. **Results**: The continuous quest for precision in dental implantology has led to the introduction of photogrammetry, which is now considered the most accurate technique for the digital scanning of dental implants. Photogrammetry has demonstrated superior results compared to those obtained using the analog technique. **Conclusions**: The aim of this case report is to provide an overview of analog techniques, digital intraoral optical scanning, and photogrammetry, setting the stage for the introduction of a novel technique involving a dedicated optical scan-transfer device (IPD^®^) that can be scanned with ease using IOS, either intra- or extra-orally, due to its unique design features and digital properties.

## 1. Introduction

Analog impressions have been the gold standard in dental implantology for many years. This traditional method involves the use of impression materials, such as polyvinyl siloxane, to register the implant position. The impression is then used to fabricate a physical model, which is used to design and manufacture a dental prosthesis. While analog impressions have been reliable, they are not without drawbacks. The process can be uncomfortable for patients and is prone to errors such as distortion, improper seating, and material shrinkage. Additionally, the time-consuming nature of analog techniques and the potential for inaccuracies at various stages have prompted the dental community to seek digital alternatives [1,2].

Digital IOS represents a significant leap forward from analog techniques. This method employs advanced optical devices to capture a 3D digital impression of the patient’s dentition and soft tissues directly within the mouth. In the case of dental implants, scanbodies are used to capture accurate 3D impressions of the implant fixtures. The primary advantages of digital intraoral scanning include enhanced patient comfort, reduced chair time, cost-effectiveness, and the immediate availability of digital data for further processing.

The high levels of trueness and precision associated with digital IOS stem from the improved physical properties of modern scanning devices. These devices use structured light or laser technology to generate detailed 3D models, which are then utilized for designing and fabricating dental prostheses with remarkable clinical results.

“Trueness and precision” are the two parameters that are used to measure the accuracy of IOS. Trueness refers to the closeness of agreement between a measured value and the actual geometry of the scanned object. Trueness measures the degree of agreement between the scan and the actual object, while precision refers to the repeatability and consistency of scan measurements. This is normally determined by comparing a reference master cast with a digitized model. Precision, on the other hand, measures the extent of variations between repeated tests [3]. Additionally, in accordance with the ISO standard for accuracy definition (ISO 5725-1:2023), accuracy is defined as the combination of trueness and precision. Trueness refers to the closeness of agreement between the average value of a large number of test results and the true or accepted reference value, while precision describes the closeness of agreement between independent test results under stipulated conditions (ISO 5725-1:2023(en), International Organization for Standardization, 2023) [4]. In this paper, the term “accuracy” is used to describe trueness and precision in the context of IOS.

Despite its advantages, IOS has limitations. Factors that affect the trueness and precision of IOS include various parameters such as scanner selection, operator experience and skill, calibration, the patient’s oral anatomy, and scanning conditions. The scanning technique, sequence, distance, and angulation are shown to play a role. Research evidence has shown that re-scanning or cut-out scanning could influence the accuracy of the digital workflow. IOS design features such as smaller camera tips, fast scanning speeds, and specific scanning patterns have been shown to affect results [3].

The scan location (anterior vs. posterior) can also be a factor. Pellitteri et al. [5] investigated the impact of different intraoral locations on the accuracy of IOS compared with the conventional polyvinyl siloxane (PVS) impression technique. Discrepancies in the range of 100–200 μm between digital and conventional impressions were reported, with significant imprecision in the molar area of both the mandible and maxilla.

Scanning large regions, particularly in cases involving multiple implants or fully edentulous arches, can introduce inaccuracies due to the absence of stable reference points and the potential for distortions. Zimmermann et al. investigated the effect of full or partial-arch IOS and demonstrated decreased values of trueness and precision in the case of full-arch scans compared with conventional analog impression techniques, concluding that while specific IOSs can be a viable alternative to traditional methods for partial-arches, full-arch scanning still presents challenges for IOSs [6]. Additionally, intraoral conditions such as saliva, blood, and limited access can significantly affect the quality of the scan. Chen et al. (2022) reported that wet conditions and liquid biological materials significantly impaired scanning accuracy in terms of both trueness and precision [7]. The evidence also points out the influence of the implant scanbody geometry, the bevel location, position, and characteristics on the trueness and precision of IOS. Longer inter-implant distances caused a significant decrease in accuracy [8]. Another study demonstrated that the bevel position, the inter-implant distance, and the position of implants near the point where scanning finished caused inaccuracies [3,9]. Ashraf et al. (2023) found that linking the scan bodies had a significant positive improvement on trueness and precision [10]. Inaccurate scans can lead to restoration misfit, screw loosening, or fractures, as well as compromised periodontal health that could result in long term complications such as restoration failure and/or peri-implant infections [11,12,13].

Recent advancements in digital impression methods have increasingly focused on improving the clinical accuracy of fit of full-arch implant-supported prostheses (Table 1). Clozza et al. (2024) described an innovative clinical technique that integrates intraoral scanning with dental photogrammetry for full-arch implant restorations [14]. In their approach, intraoral scanning was used to capture detailed soft tissue and occlusal morphology in real time, while photogrammetry was employed to accurately record the three-dimensional positions of the implants via triangulation from multiple high-resolution photographs. This hybrid method was specifically designed to mitigate the cumulative stitching errors that often occur with intraoral scanners when capturing large spans, such as edentulous arches. Although this technique required a more complex workflow—with dedicated photographic acquisition and subsequent image processing—it effectively leveraged the strengths of both modalities to enhance overall accuracy.

Joensahakij et al. (2024), [13] in a systematic review, compared the accuracy of conventional impression techniques with digital methods, including both intraoral scanning and photogrammetry, for full-arch implant-supported prostheses (Joensahakij et al., 2024; PMID: 39134687). Their review highlighted that digital impression methods generally offer trueness and precision comparable to, or in some cases better than, conventional techniques. Notably, the review underscores the potential of photogrammetry to overcome the limitations of intraoral scanners, particularly the error accumulation inherent in long-span or edentulous situations. However, the authors also note a degree of heterogeneity in the literature, emphasizing the need for standardized protocols across studies.

Building on these findings, a novel optical scan-transfer device (IPD^®^ ST) represents a further evolution in digital workflows for full-arch implant reconstruction. In our approach, scan-transfer abutments are placed on implants at the time of surgery. These STs are then rigidly linked with dental resin to create a stable “jig” that is removed from the oral cavity and scanned extra-orally using an intraoral scanner. By shifting the scanning process outside the challenging intraoral environment, where moisture, bleeding, and limited access can compromise scan quality—this technique minimizes cumulative errors. In addition, this extraoral scanning approach circumvents the need for the stitching process that is inherent to conventional IOS methods, thereby improving both trueness and precision.

Photogrammetry (PG), a technique traditionally used in fields such as cartography and engineering, has recently been adapted for dental applications. Photogrammetry has demonstrated impressive outcomes in digital scanning; however, direct comparisons with emerging techniques such as hybrid scanning approaches are still needed. This method involves capturing a series of high-resolution photographs from multiple angles and using specialized software to reconstruct a 3D model.

In dental implantology, photogrammetry is used to record the three-dimensional positions of implants in edentulous arches and has been shown to offer the highest results in terms of trueness and precision, particularly in full-arch cases involving multiple implants [13].

The main advantage of photogrammetry is its ability to capture detailed 3D data without being affected by intraoral conditions. The technique allows for the correct alignment of implant positions, even in challenging clinical scenarios. However, photogrammetry requires sophisticated software and costly equipment. The process can be more time-consuming compared with direct intraoral scanning. Furthermore, an additional intraoral IOS is still required alongside PG to capture the soft tissue profile. More recently, a new technique termed grammetry, which uses modified scanbodies with geometric shapes, was proposed as an alternative to PG (Roe: ROE Dental Laboratory. Available at: https://www.roedentallab.com/products/implants/full-arch-fixed-options/grammetry/ (accessed on 8 November 2024). However, this technique uses complex scanbodies (SBs) that require careful intraoral positioning and fixation with inherent difficulties and costs.

## 2. Case Presentation: Introducing a Novel Scan-Transfer Device for a Full-Arch Digital Workflow in the Edentulous Maxilla

**Figure 1:**
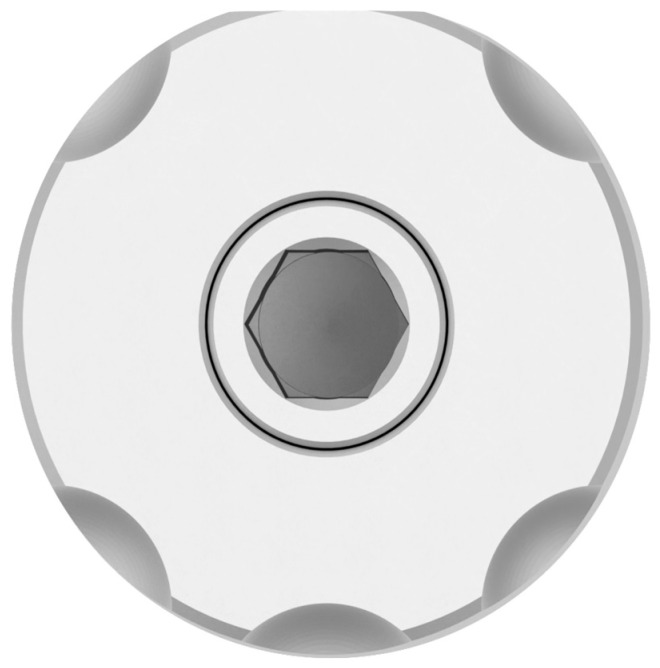
Scanning the IPD^®^ Scanbody.

**Figure 2:**
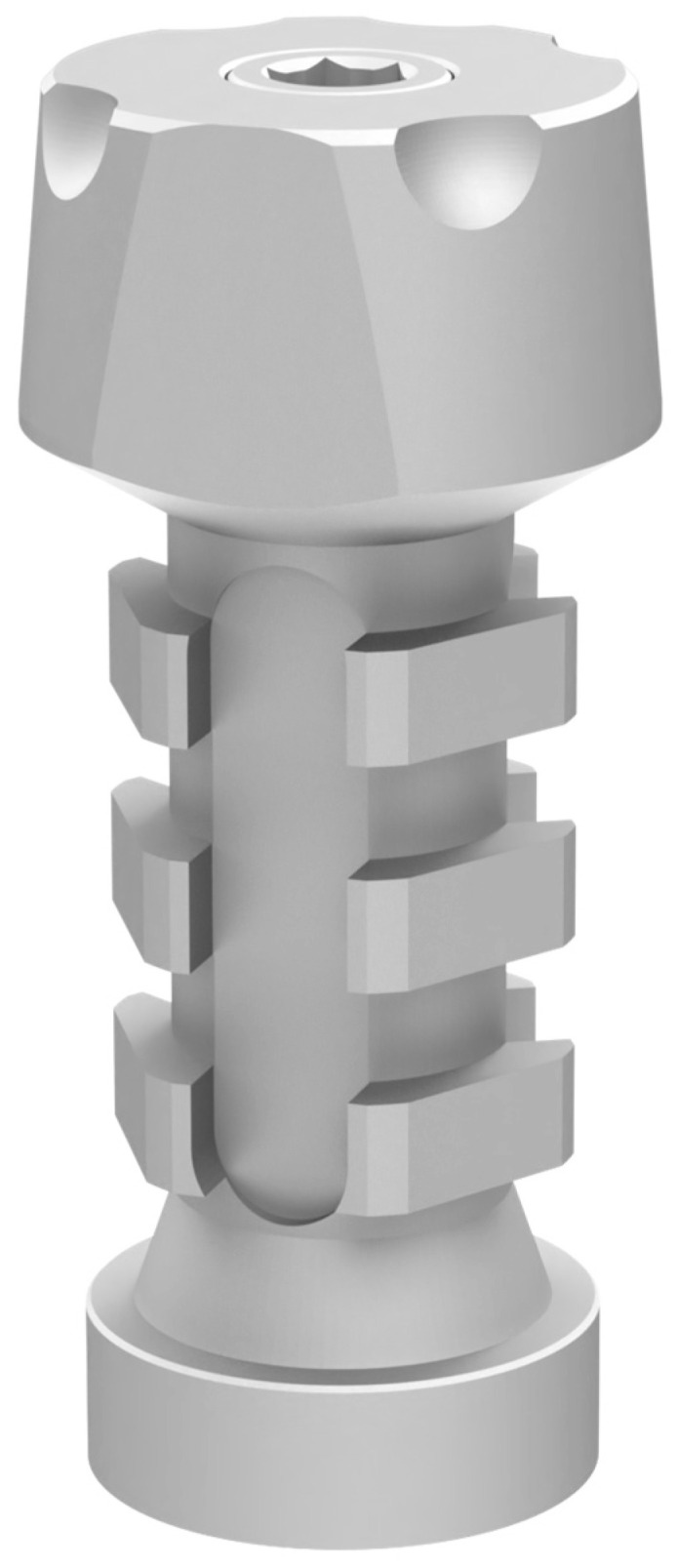
IPD^®^ scan-transfer abutment (ST) for multi-unit abutments (MUAs).

**Figure 3:**
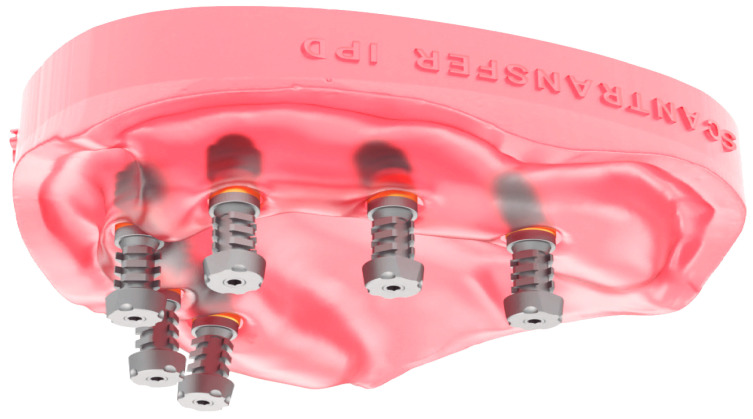
IPD^®^ scan-transfer abutments (STs) are specifically designed to fit onto the standard diameter 4.8 conical MUA platform of different makes. In extended-arch or full-arch cases, the STs are linked together using acrylic resin before being scanned chair-side outside the mouth.

**Figure 4:**
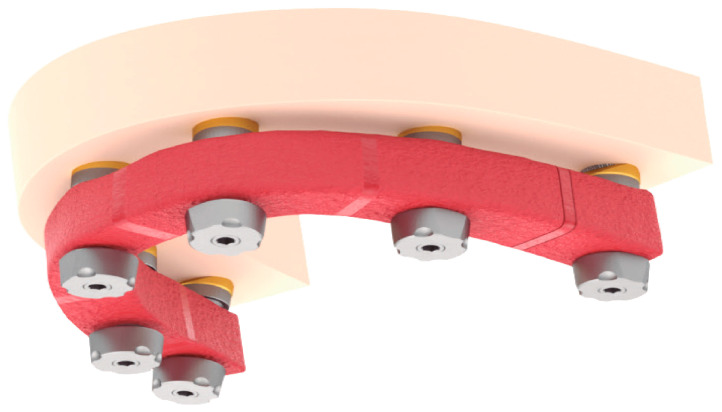
Improving scan device stability by linking IPD^®^ STs: linking STs together not only enhances stability but also provides 3D architecture and reference points between the scan bodies to help the IOS navigate its way around the mouth.

**Figure 5:**
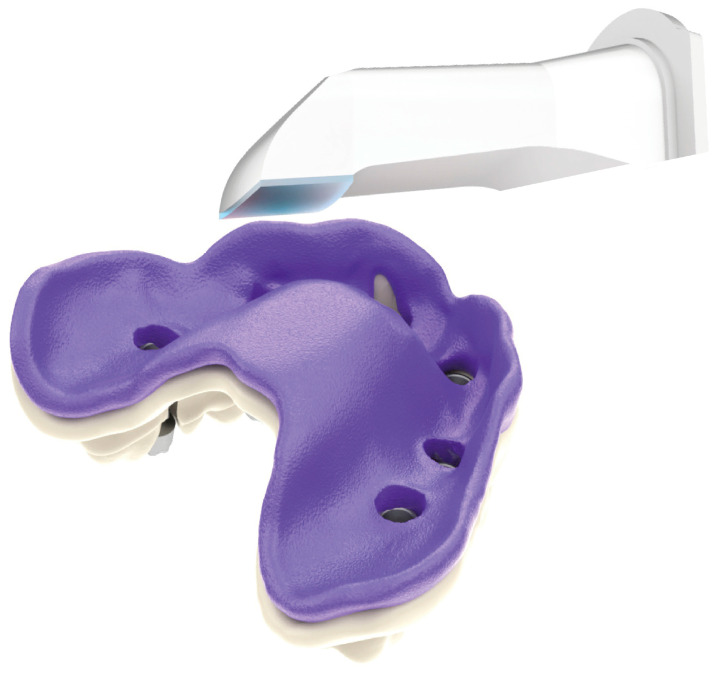
Extra-oral scanning with putty pick-up impression.

**Figure 6:**
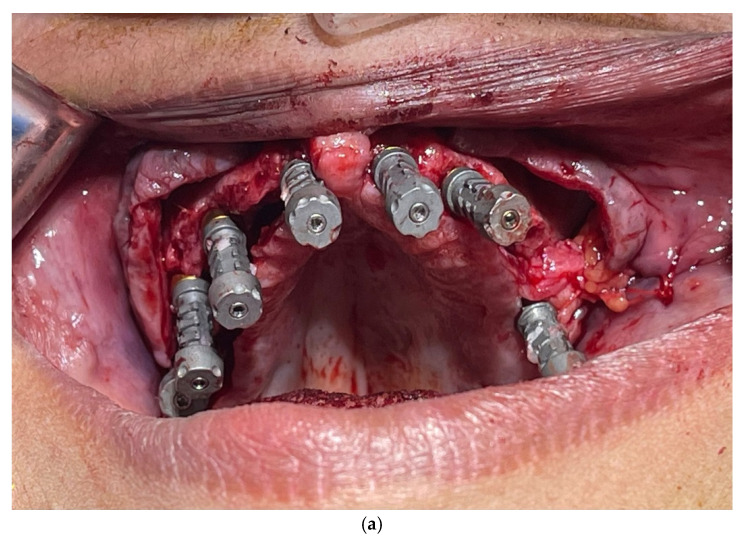

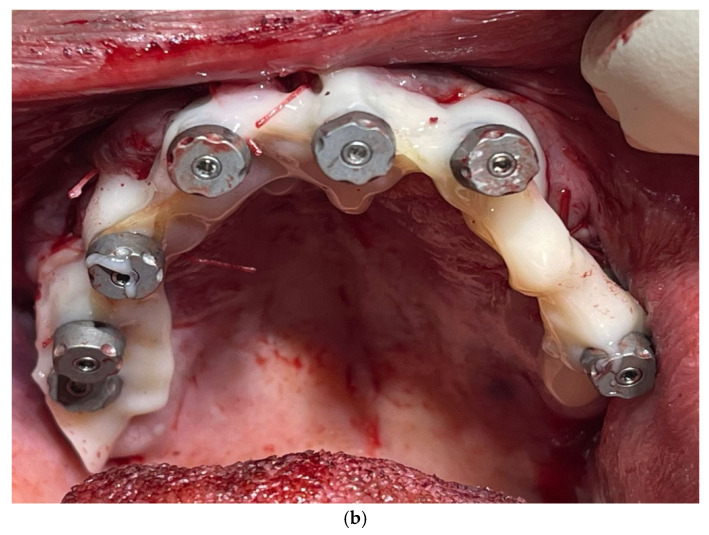
(**a**) IPD^®^ scan-transfers connected to multi-unit abutments for extra-oral scanning. (**b**) IPD^®^ scan-transfer abutments (STs) linked with dental resin for extra-oral chairside scanning.

**Figure 7:**
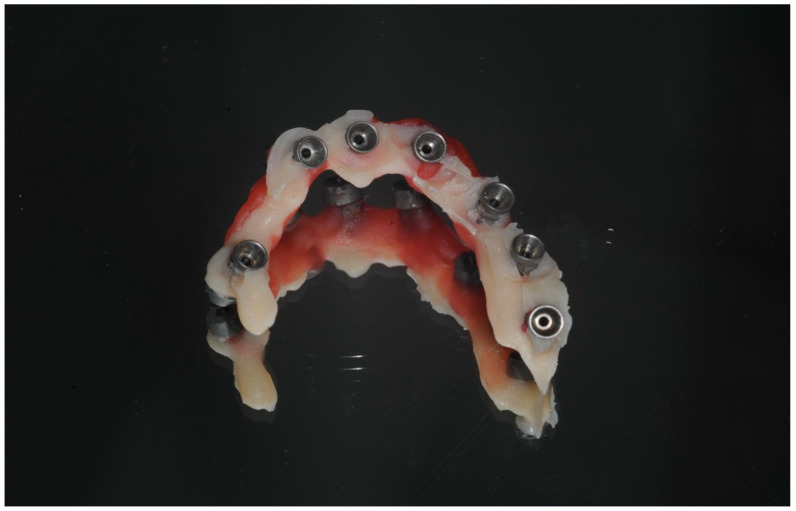
A view of the fitting surface of the acrylic jig with IPD^®^ STs used for extra-oral chairside scanning.

This study was conducted in accordance with ethical principles, including the World Medical Association Declaration of Helsinki. The patient agreed with the treatment plan and provided informed consent. Building on the strengths and addressing the limitations of existing methods, this paper introduces a novel technique for digital optical scanning using a specifically manufactured optical scan-transfer abutment (ST) by IPD^®^. This new scan-transfer device was designed to be easily scanned outside the mouth, thus eliminating the significant disadvantages associated with IOS when carried out in the presence of bleeding (Figure 1, Figure 2 and Figure 3). There is no need to scan the shank of the STs, which feature retention grooves that allow adjacent STs to be rigidly linked with dental resin (or silicone impression material) to create a stable splint which facilitates easier scanning either in or outside the mouth (Figure 1, Figure 2, Figure 3, Figure 4, Figure 5, Figure 6 and Figure 7).

This device has a dual function, in that it can also be used as a post for conventional analog impression techniques.

The current technique involves placing optical scan-transfer abutments (STs) on the implants immediately at the time of surgery. The STs, which are tailored for optimal optical properties and precise fit, are then linked together with dental resin and removed from the mouth to be scanned outside the oral cavity with any IOS device. This approach minimizes the impact of intraoral conditions and provides highly accurate digital reference points of the implant positions.

Details of Materials, scan-transfer abutments and analogs used in this case study:

Brand: IPD^®^ (Only available from Online-Dental.uk in the UK).

City of Manufacture: Barcelona (IPD2004, C. Rosa dels Vents, nº 9–15, 08,338 Premià de Dalt, Barcelona).

Country of manufacture: Spain.

Software used: Exocad DentalCAD 3.2 Elefsina.

Bridge milled from G cam from Graphenano^®^ dental.

Finished with Optiglaze^®^ from GC dental.

Ti-bases cemented using Panavia V5 opaque.

Model printed with Optiprint from Dentona on an Asiga Max 2.

### The Technique

Patient Information:

Age/Gender: 50-year-old female.

Medical History: No significant medical conditions.

Chief Complaint: Functional difficulties with a full maxillary denture worn for five years.

Clinical Assessment:

The patient presented with a fully edentulous maxilla, expressing dissatisfaction with her current removable denture due to poor stability and function. A fixed full-arch prosthesis on implants was determined to be the most suitable treatment option.

Treatment Plan:

The treatment was planned to use a 3D digital workflow with 3Shape Implant studio^®^. A surgical drill guide was designed and 3D-printed using a Sprintray^®^ printer and Keystone^®^ surgical guide resin. The main focus of the treatment was to provide delayed–immediate loading of the full-arch bridge within a couple of days of placement of the implants, as the patient lived abroad and wanted to return home sooner. 

The authors chose the current protocol, they refer to as Tomorrow’s Teeth, over alternative approaches like immediate denture conversion or fully guided surgery (Same Day Teeth concept). This decision was based on improved predictability, efficiency, and superior aesthetic and occlusal outcomes achieved with a laboratory-produced provisional bridge compared with, for example denture conversions. By constructing the bridge on the actual implants after placement, the process ensures greater precision and overall treatment success.

A novel IPD^®^ digital workflow utilizing optical scan-transfer abutments (STs) (Figure 1 and Figure 2) was selected to ensure high precision in implant-restorative optical scanning while addressing the challenges of intraoral scanning in surgical environments with moisture and bleeding.

Implant Placement and Abutment Selection:

Multiple dental implants were placed in the maxilla under local anesthesia and conscious sedation. Multi-unit abutments (MUAs) were selected based on angulation and collar height (Figure 2).

Extraoral Digital Scanning: The first stage of the digital workflow involved using IPD^®^ STs intra-operatively. After implant placement, the appropriate multi-unit abutments, selected for their angulation and collar height, were fitted onto the implants. The STs were then connected to the MUAs, with their screwheads carefully checked for full seating to ensure a correct fit (Figure 5 and Figure 6). The surgical flap was approximated and lightly sutured with resorbable suture material (Vicryl^®^ 3.0). The STs were then linked together to construct a “jig” with self-cured dental resin (e.g., Bredent^®^ QuResin) (Figure 4 and Figure 6). To capture the soft tissue profile surrounding the MUAs, putty impression material was applied around the gingival surfaces of the jig, ensuring that the heads of the STs remained free of the impression material or dental resin to facilitate scanning after the jig had been removed from the mouth (Figure 7). Plastic caps are available to protect the heads of the STs when linking them with dental resin.

The second stage of the digital workflow, after linking the STs, involved removing the acrylic jig and performing extra-oral, chairside scanning of all surfaces of the jig (Figure 5 and Figure 7). This process is usually much more straightforward and less time-consuming compared with intraoral scanning, especially when it is performed at the time of implant placement surgery when the field is full of moisture, saliva, and blood. Studies suggest that linking scan bodies improves scan properties by providing stable reference points, though further experimental validation is necessary to confirm these findings in full-arch cases [1,10].

**Figure 8:**
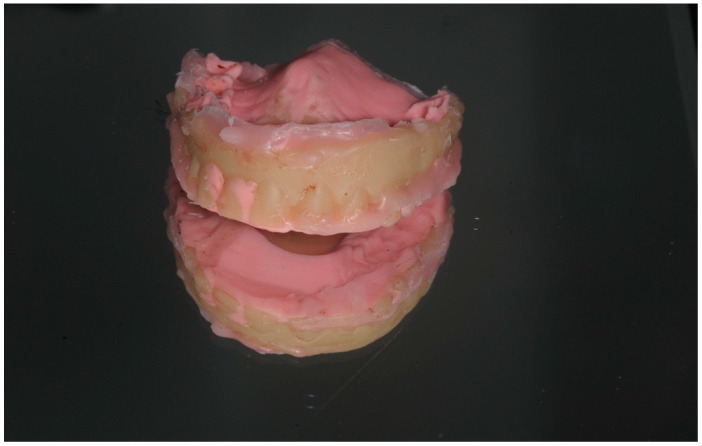
Bite registration process using digital workflow (DWF).

**Figure 9:**
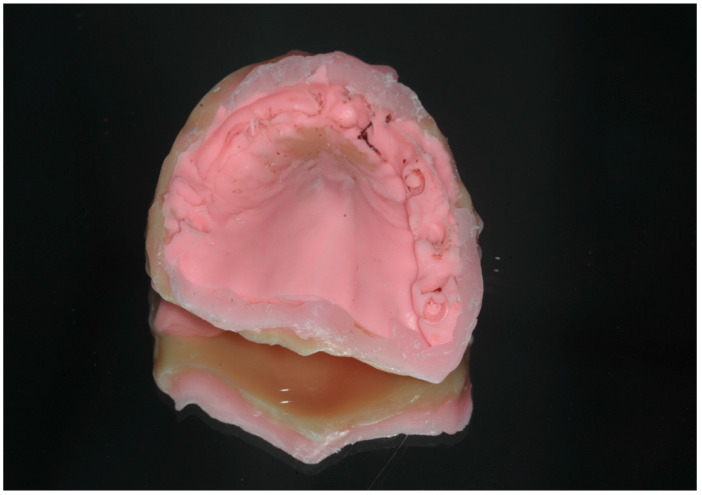
The fitting surface of the bite registration device used in the DWF.

**Figure 10:**
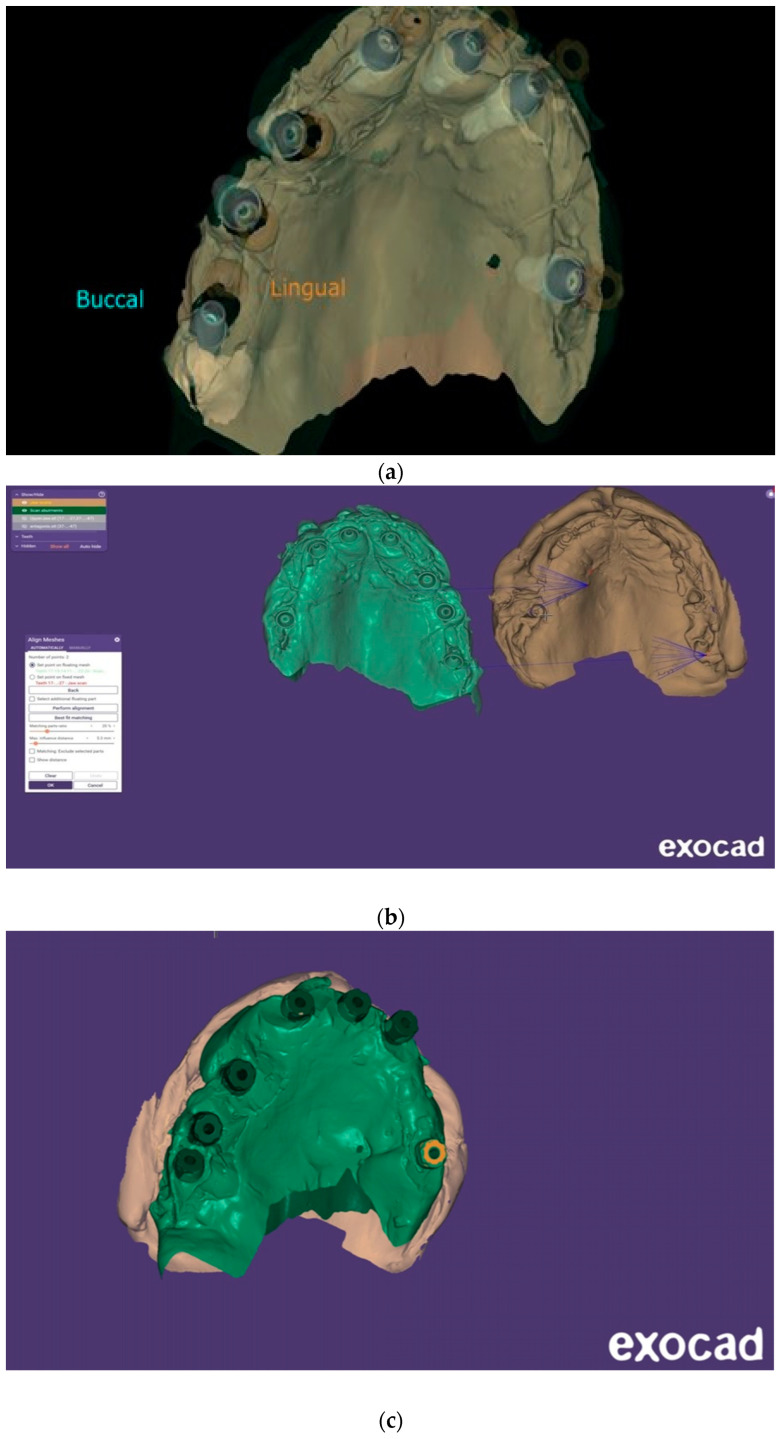
(**a**) Exocad DentalCAD 3.2 Elefsina rendered image of the IPD^®^ STs. (Lab work G Jones, and S Tilaei, Creative Implant Solutions, Bolton, UK). (**b**) A view showing the process of merging ST scan data with scan data obtained from the bite registration device using Design Web Format (DWF). (CIS). (**c**) The ST scan data were merged with scan data obtained from the bite registration device obtained by chairside scanning.

**Figure 11:**
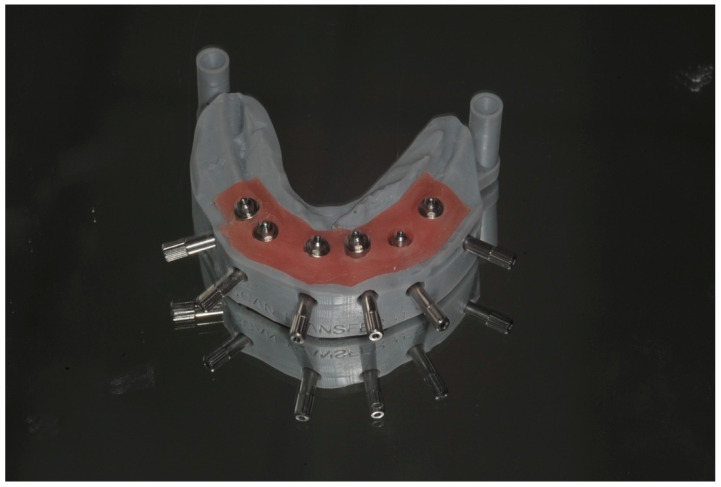
3D model printing after scanning IPD^®^ scan-transfer abutments (STs).

**Figure 12:**
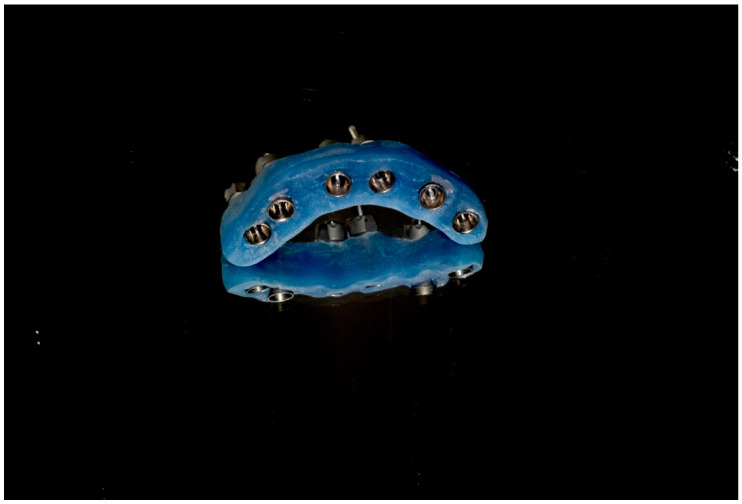
A verification jig produced on the 3D printed model can be used to check the clinical accuracy of fit intraorally before producing the prosthesis (courtesy of Nicole Straw, Go Digital Ltd., Buckinghamshire, UK).

The final intraoral prosthodontic stage included bite registration using a copy denture technique. This involved using a prefabricated copy denture device constructed from the diagnostic denture (Figure 8 and Figure 9). The device was lined with dental putty to capture the soft tissue profile of the intaglio surface and was used to accurately register the bite. It was then removed from the mouth and all surfaces of this registration device were scanned extra-orally using an intraoral scanner (IOS) (Figure 5 and Figure 7). Finally, the opposing arch was scanned, and all scans were digitally uploaded to the laboratory for processing using suitable Computer-Aided Design/Computer-Aided Manufacturing (CAD/CAM) software (Exocad^®^) DentalCAD 3.2 Elefsina (Figure 10, Figure 11 and Figure 12). This digital protocol allowed the dental laboratory to commence the design and milling of the provisional full-arch bridge within an hour of the placement of the implants, and thus significantly speeding up the process compared with analog techniques. The laboratory processing time for the digital design of the bridge was approximately 4 h when the bridge was 3D-milled using a re-enforced polymethyl methacrylate PMMA disc (Graphenano Dental^®^). MUA female sleeves were fitted to the PMMA bridge after the milling process.

**Figure 13:**
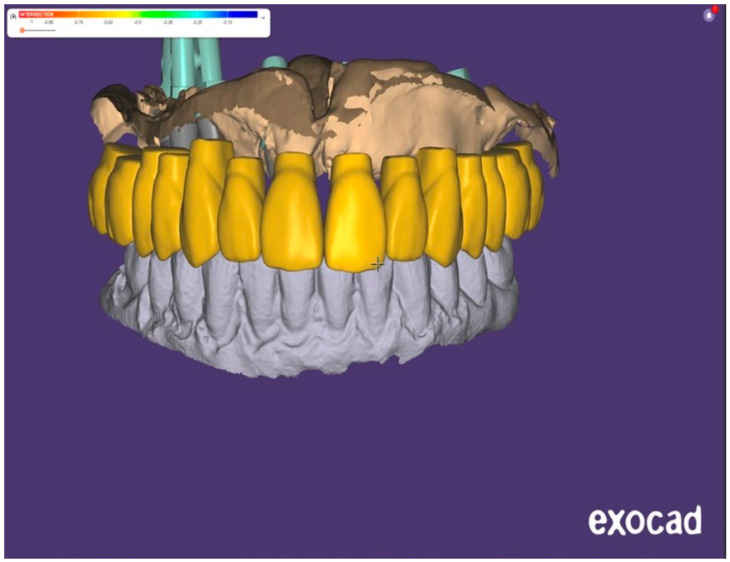
Exocad^®^ DentalCAD 3.2 Elefsina virtual design of the full--arch prosthesis after importing the scan data obtained extra-orally using IPD^®^ STs.

**Figure 14:**
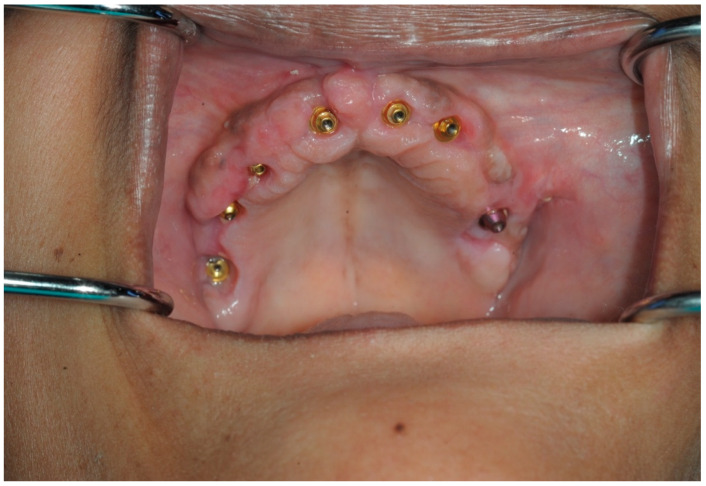
An intraoral view of a full-arch implant reconstruction using Anyridge^®^ Megagen MUAs restored using the novel digital workflow described in this article.

The prosthesis can be produced entirely through a digital workflow or alternatively by printing a 3D model to verify the prosthesis fit (Figure 11). A verification jig may also be used for this purpose (Figure 8, Figure 9, Figure 10, Figure 11, Figure 12, Figure 13 and Figure 14). The prosthesis can be fabricated with or without titanium bases (e.g., ti-bases); if produced without ti-bases, it is fitted directly onto the MUAs using special retention screws.

**Figure 15:**
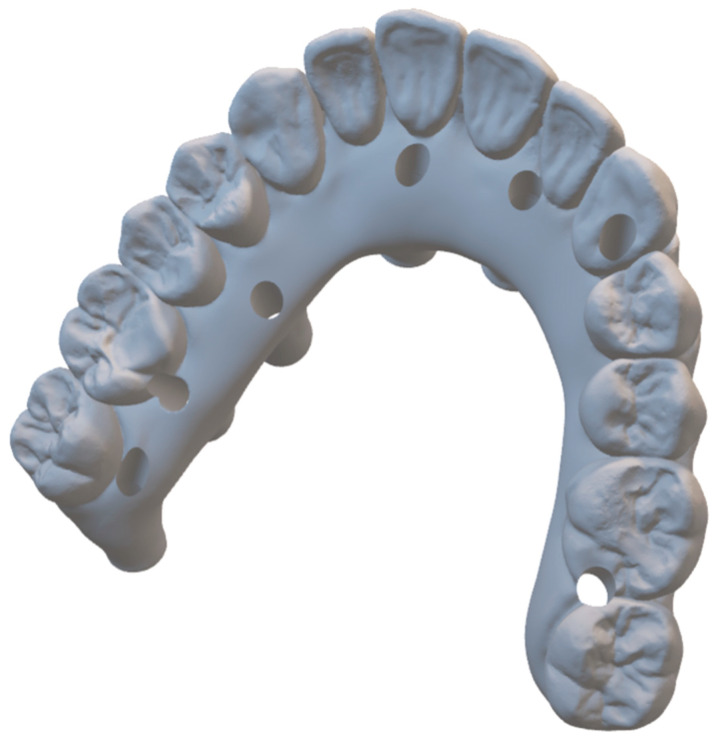
An STL file of the virtually designed bridge ready for 3D milling or 3D printing in PMMA or dental resin, respectively.

**Figure 16:**
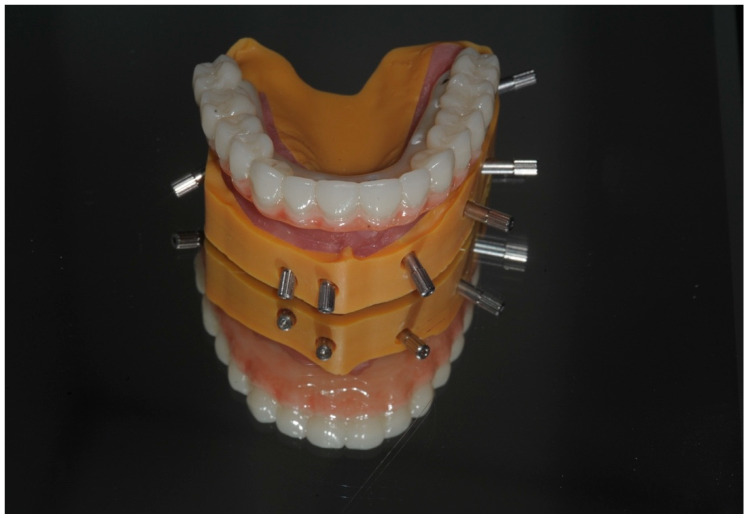
A CAD-designed bridge can be tried on the 3D-printed IPD^®^ model to check the prosthetic fit.

**Figure 17:**
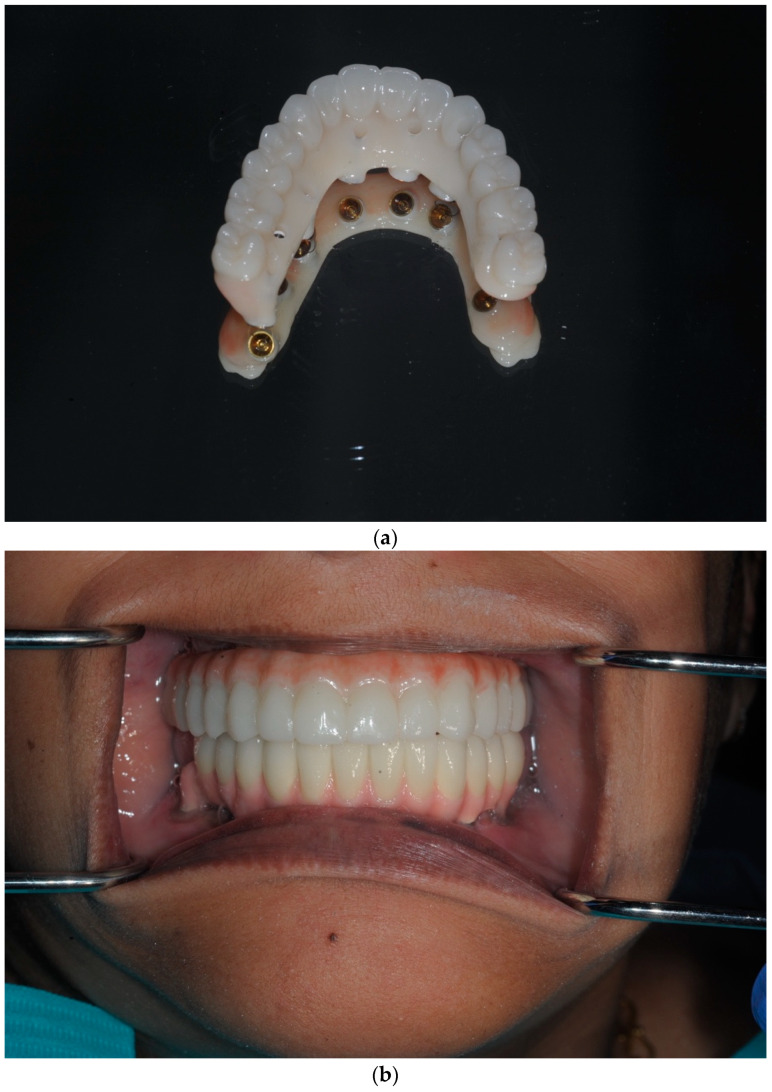

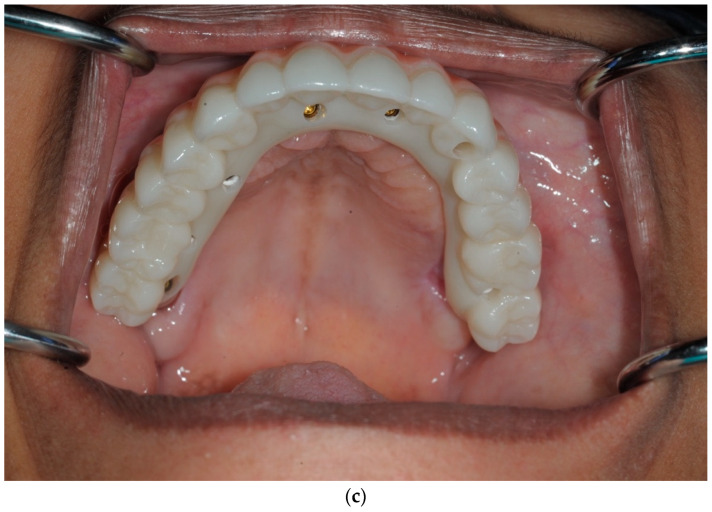
(**a**) The provisional prosthesis fitted in the current case was produced using the IPD^®^ digital workflow described above. The provisional bridge can be produced with or without titanium sleeves. When produced without titanium sleeves, the bridge can be fitted directly onto the multi-unit abutments (MUAs). (**b**) The provisional bridge in-situ, opposing a full-arch mandibular bridge. Note the occlusal integration. (**c**) An occlusal view of the provisional bridge produced using the novel IPD^®^ DWF.

The prototype prosthesis was fitted on the 2nd post-operative day, which achieved an excellent clinical fit and occlusal integration whilst also satisfying the patient’s aesthetic requirements (Figure 15, Figure 16 and Figure 17).

This 3D digital approach, undertaken in the current case, significantly improved clinical efficiency and patient comfort compared with alternative approaches for full-arch loading. By eliminating intraoral scanning challenges during surgery, the workflow reduced the clinical chairside time and improved the duration and efficiency of the laboratory stages. The patient reported high satisfaction with the new fixed prosthesis, which achieved good stability, function, and aesthetics.

This case highlights the efficacy of extraoral scanning with optical scan-transfer abutments, demonstrating its potential as a clinically acceptable and efficient digital solution for full-arch implant rehabilitation.

The IPD^®^ scan-transfer abutments (STs) are designed to be scanned only around their heads, making it unnecessary to scan the shank. This feature allows for extra-oral scanning once the STs are removed from the mouth using a putty impression or an acrylic resin jig that links the adjacent STs together. The scanning is then performed chairside using an intraoral scanner (IOS) and uploaded digitally to the laboratory (Figure 1 and Figure 2).

The IPD^®^ STs are specifically designed to fit onto a multi-unit abutment (MUA) of different makes. In extended-arch or full-arch cases, the STs are linked together using acrylic resin before being scanned chairside outside the mouth (Figure 3 and Figure 4).

Once the STs are rigidly connected, they can be captured with a conventional putty impression, ensuring that the peri-implant soft tissue profile is included. Alternatively, silicone impression material can be pressed under the acrylic jig to capture the soft tissue profile before removing the jig. In this case, a full conventional impression is unnecessary, as the rigidly linked STs with the acrylic jig provide the necessary landmarks for scanning (see Figure 5, Figure 6 and Figure 7).

After unscrewing the STs, the putty pick-up impression is removed and scanned chairside, extra-orally, capturing all surfaces. With this digital technique, no intraoral scanning is required.

In this case, the IPD^®^ STs were connected to the multi-unit abutments (MUAs) on Anyridge^®^ Megagen implants. Note that the ST screwheads were fully fitted and were flush with the top of the STs, a feature designed to provide visual confirmation of their correct seating.

The STs were linked together using Bredent^®^ QuResin. The putty impression material was applied around the gingival surfaces of the jig to capture the soft tissue profile surrounding the MUAs. It is important to ensure that the heads of the STs remain free of acrylic to allow for accurate scanning once the jig is removed from the mouth. Protective plastic caps are provided for this purpose.

This view shows the fitting surface of the acrylic jig incorporating the IPD^®^ STs. The impression putty was pressed beneath the jig to capture the peri-implant soft tissue profile surrounding the MUAs. With this technique, a separate intraoral analog or digital impression was not required.

Bite registration was performed using a prefabricated copy denture made from acrylic. The fitting surface of the denture was lined with impression putty to capture the heads of the MUAs. This aids the technician in accurately aligning the bite registration on the virtual model using CAD software, such as Exocad^®^ DentalCAD 3.2 Elefsina (Figure 8).

The fitting surface of the bite registration device was lined with impression putty or a light-body silicone impression material. The device can be scanned on both surfaces chairside using an intraoral scanner (IOS) and then imported into CAD software, such as Exocad^®^ DentalCAD 3.2 Elefsina for further processing; see Figure 9 and Figure 10.

After scanning the IPD^®^ ST with a chairside IOS device, the digital data were imported into CAD software, such as Exocad^®^ DentalCAD 3.2 Elefsina. A master model was then printed using the IPD^®^ libraries. IPD^®^ offers a secure analog system with improved stability of the printed model with two fixation screws: one base screw and a transfer screw which ensures the correct height of the analog in the model and prevents any lateral movement (Figure 11, Figure 12 and Figure 13). Alternatively, the prosthesis can be designed and manufactured entirely in a virtual environment, without a 3D-printed model, using compatible CAD/CAM software. IPD^®^ provides libraries for commonly used systems, including 3Shape^®^, Dental Wings^®^, and Exocad DentalCAD 3.2 Elefsina (Figure 14, Figure 15, Figure 16 and Figure 17).

## 3. Discussion

The advent of digital workflows and intraoral scanning (IOS) has revolutionized modern dentistry, particularly for patients undergoing dental implant therapy. Compared to traditional analog techniques, digital workflows offer numerous advantages, including greater efficiency, reduced chairside time, and improved cost-effectiveness [16,17]. The use of IOS enables a faster and more streamlined process, benefiting both clinicians and patients by minimizing the treatment time and reducing the number of required appointments.

One of the key benefits of digital workflows is their reliability in producing successful clinical outcomes in short-span and partially edentulous implant cases. Research consistently shows that IOS performs comparably to analog techniques in these situations. However, challenges arise when IOS is applied to more complex, large-span, full-arch implant cases. In such cases, accuracy—defined as trueness and precision—tends to decrease, particularly as inter-implant distances increase. Several factors contribute to this decline, including the absence of stable reference points, implant angulation, and their positioning within the oral cavity [18,19]. Additionally, the presence of moisture and blood further complicates the scanning process, often necessitating multiple rescans and prolonging the procedure.

To overcome these limitations, innovative solutions such as novel scan-transfer (ST) devices have been introduced. The IPD^®^ scan-transfer device is specifically designed to mitigate factors that compromise IOS accuracy in complex implant cases [20,21]. By enabling the scanning of implant positions outside the oral cavity, the IPD^®^ ST device reduces the impact of moisture, blood, and challenging implant angulations—common obstacles in traditional intraoral scanning. This external scanning approach not only shortens scanning sessions but also minimizes errors caused by repeated rescanning [22].

Furthermore, the scan-transfer technique incorporates splinted STs, which are connected using dental resin to maintain consistent reference points between implant scan bodies. This significantly enhances the scanning process, allowing the digital workflow to be effectively employed even in full-arch cases—providing a much-needed solution to previous limitations [22,23].

Beyond improved scanning accuracy, the digital workflow offers several other notable benefits. One major advantage is cost-effectiveness, as it significantly reduces laboratory costs compared to conventional methods [24,25]. Additionally, the ease of integration and the ability to perform extraoral scanning using existing IOS scanners streamline the workflow, making it more user-friendly for both clinicians and dental technicians.

The IPD^®^ ST device also enables the three-dimensional (3D) capture of both implant scan-transfers and the surrounding soft tissue profile, providing a more comprehensive and detailed impression of the patient’s oral anatomy. This dual-purpose functionality allows it to serve as both a scan-transfer body and a verification jig, ensuring the correct fit of the final restoration. Moreover, it can be used for traditional analog impressions, offering versatility in cases where digital techniques may not be suitable or preferred [26].

Despite its advantages, the focus on the IPD^®^ device may limit generalizability, as variations in intraoral scanner brands and different clinical scenarios could impact its effectiveness. Further independent studies are necessary to assess its broader applicability. While previous studies and theoretical foundations support the utility of the IPD^®^ technique, comparative experimental research is required to validate its precision against photogrammetry and conventional analog impressions.

Future research should include controlled trials to evaluate clinical performance, establish standardized accuracy metrics for IOS in complex restorations, and conduct randomized controlled trials comparing digital workflows with traditional impression techniques and photogrammetry.

Although digital workflows are often described as cost-effective, comprehensive cost–benefit analyses and patient satisfaction studies are needed to substantiate these claims.

### Limitations

Despite the promising results achieved with the novel optical scan-transfer device (IPD^®^), this technique has only been demonstrated in a single case report. Larger-scale clinical studies and controlled trials are needed to confirm its reproducibility and assess long-term clinical outcomes.

The extraoral scanning process involves splinting scan-transfer abutments with dental resin and subsequently removing them from the mouth, introducing additional steps that are operator-dependent. Variability in technique could impact overall accuracy.

Additionally, the method relies on the dedicated IPD^®^ device and its compatibility with specific intraoral scanners. Differences in scanner technology and design across various systems may influence performance and limit the generalizability of this approach.

## 4. Conclusions

The evolution of dental implantology from analog impressions to digital workflows has significantly improved the efficiency of implant placement as well as prosthetic fabrication. While digital intraoral optical scanning has advanced the field, photogrammetry has set a new benchmark. However, the latter technique has its significant drawbacks, including high initial cost of investment.

In this case study, a simple novel scan-transfer device demonstrated the benefits of using an extraoral digital workflow, addressing the limitations of intraoral scanning in complex surgical conditions. This method facilitated the satisfactory delivery of a fixed, full-arch implant-supported prosthesis, with clinically verified prosthetic fit, occlusal integration, and good aesthetic outcomes.

However, further research with larger cohorts is necessary to validate the accuracy with reference to the trueness, precision, and clinical efficacy of this innovative approach.

## Figures and Tables

**Table 1:** Comparison of Photogrammetry, intraoral scanning and conventional techniques.

Technique	Principle	(Trueness and Precision)	Advantages	Disadvantages	References
Conventional Impression Technique	Elastomeric impression (e.g., splinted open-tray) with cast fabrication	Generally high accuracy if performed properly; minimal cumulative error in full-arch cases but technique-dependent	Proven clinical performance; well-documented reliability	Time-consuming, uncomfortable for patients, and sensitive to material dimensional changes	[1,11]
Intraoral Scanning (IOS)	Direct digital capture using structured light/laser scanning with image stitching	Acceptable accuracy for short spans; however, error accumulation may occur in complete-arch or edentulous situations due to stitching challenges	Fast, patient-friendly, immediate digital output, integrated into digital workflows	Susceptible to cumulative stitching errors, especially in long-span or edentulous arches	[12]
Photogrammetry	Reconstruction from multiple 2D photographs via triangulation	Studies indicate very high trueness and precision with lower 3D discrepancies in complete-arch implant impressions; less affected by cumulative error	Cost-effective, highly accurate for full-arch applications, and accessible for training purposes	More time-intensive (requires multiple photographs and post-processing), generally used with plaster models. Higher cost of scan equipment	[11,13,15]

## Data Availability

Data are contained within the article.
